# Hierarchically Porous Polypyrrole Foams Contained Ordered Polypyrrole Nanowire Arrays for Multifunctional Electromagnetic Interference Shielding and Dynamic Infrared Stealth

**DOI:** 10.1007/s40820-024-01588-x

**Published:** 2024-12-26

**Authors:** Yu-long Liu, Ting-yu Zhu, Qin Wang, Zi-jie Huang, De-xiang Sun, Jing-hui Yang, Xiao-dong Qi, Yong Wang

**Affiliations:** School of Chemistry, Key Laboratory of Advanced Technologies of Materials (Ministry of Education), Southwest Jiaotong University, Chengdu, 610031 People’s Republic of China

**Keywords:** Polypyrrole nanowire arrays, Hierarchical foam, Hydrophobicity, Infrared stealth, Electromagnetic interference shielding

## Abstract

As modern communication and detection technologies advance at a swift pace, multifunctional electromagnetic interference (EMI) shielding materials with active/positive infrared stealth, hydrophobicity, and electric-thermal conversion ability have received extensive attention. Meeting the aforesaid requirements simultaneously remains a huge challenge. In this research, the melamine foam (MF)/polypyrrole (PPy) nanowire arrays (MF@PPy) were fabricated via one-step electrochemical polymerization. The hierarchical MF@PPy foam was composed of three-dimensional PPy micro-skeleton and ordered PPy nanowire arrays. Due to the upwardly grown PPy nanowire arrays, the MF@PPy foam possessed good hydrophobicity ability with a water contact angle of 142.00° and outstanding stability under various harsh environments. Meanwhile, the MF@PPy foam showed excellent thermal insulation property on account of the low thermal conductivity and elongated ligament characteristic of PPy nanowire arrays. Furthermore, taking advantage of the high conductivity (128.2 S m^−1^), the MF@PPy foam exhibited rapid Joule heating under 3 V, resulting in dynamic infrared stealth and thermal camouflage effects. More importantly, the MF@PPy foam exhibited remarkable EMI shielding effectiveness values of 55.77 dB and 19,928.57 dB cm^2^ g^−1^. Strong EMI shielding was put down to the hierarchically porous PPy structure, which offered outstanding impedance matching, conduction loss, and multiple attenuations. This innovative approach provides significant insights to the development of advanced multifunctional EMI shielding foams by constructing PPy nanowire arrays, showing great applications in both military and civilian fields.

**Supplementary Information:**

The online version contains supplementary material available at 10.1007/s40820-024-01588-x.

## Highlights

- Hierarchical melamine foam (MF)/polypyrrole (PPy) nanowire arrays (MF@PPy) were fabricated by electrochemical polymerization.
- The 3D porous PPy micro-skeleton and the 1D PPy nanowire arrays imparted the MF@PPy foams with high electromagnetic interference shielding performance of 19,928.57 dB cm^2^ g^−1^ via multiple attenuations.
- The PPy nanowire arrays achieved multifunctional integration including hydrophobicity, thermal insulation, and dynamic infrared thermal camouflage properties.

**Supplementary Information:**

The online version contains supplementary material available at 10.1007/s40820-024-01588-x.

## Graphical abstract

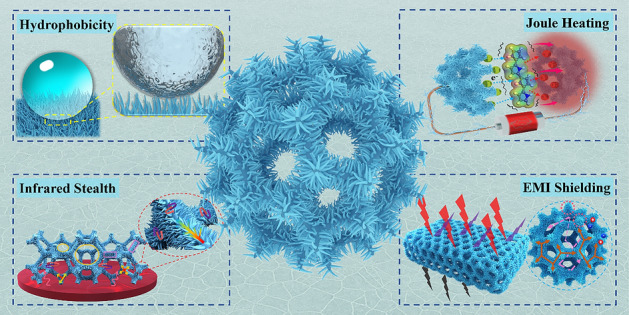

## Introduction

The vigorous evolution of the fifth-generation information technology and the proliferation of electromagnetic devices have significantly enhanced societal productivity and living standards. However, this advancement has also led to an increase in electromagnetic pollution [1, 2]. This pervasive issue has generated detrimental effects on human health and system failure. Therefore, the innovation of advanced electromagnetic interference (EMI) shielding materials has become an essential approach to address the escalating concerns associated with electromagnetic pollution [3, 4]. Conductive polymer composites (CPCs) have garnered considerable attention as exemplary EMI shielding materials, owing to lightweight, low cost, good chemical stability, and convenient processing [5–7]. Compared to conventional dense bulk EMI shielding materials, porous foams with three-dimensional (3D) skeleton can provide multiple interfaces and achieve better impedance matching, thus enabling to more effectively absorb and attenuate incident electromagnetic waves (EMWs) [8, 9].

It is well known that melamine foam (MF) has the characteristics of high porosity, lightweight, excellent resilience, and environmental friendliness, making it an excellent substrate for EMI shielding materials [10, 11]. Its inherent 3D network framework serves as an ideal template for constructing a continuous conductive pathway with low filler content [12, 13]. Some researchers have been actively exploring the fabrication of MF-based EMI shielding materials by binding/depositing electrically conductive fillers (such as MXene, graphene, silver nanowire arrays, metal–organic frameworks, etc.) onto the framework of foam [14–16]. Benefiting from the well-established 3D conductive network and inner porous structure, the MF-based materials exhibit high EMI shielding performance by multiple absorption. Furthermore, the future application of EMI shielding materials may extend into more intricate and diverse practical scenarios and fields, requiring more functions to meet the growing demand simultaneously. For instance, flexible and superhydrophobic EMI shielding materials are urgently desirable for protecting sensitive electronic devices or human health. Meanwhile, infrared stealth is an important method to hide the features of covered objects in the infrared frequencies of the EM spectrum. This puts forward higher requirements for material function integration in fields such as flexible sensing, water repellence, thermal management, and infrared stealth [7, 17, 18]. For example, Liu et al. fabricated MF/Ni flower/MXene hybrid foams via electrostatic self-assembly and dip-coating, achieving the integration of EMWs absorption, flame retardant, and infrared stealth [19]. Wang et al. prepared MF/Fe_3_O_4_/silver nanowire foams through coprecipitation and dip-coating processes, showing the advantages of good absorption-dominant EMI-shielding performance, high thermal insulation, and flame retardancy [16]. These advances are critical to meet the complex demands of multifunctional EMI-shielding materials in real-world applications. However, the conductive fillers are usually deposited on MF by direct spraying, dip-coating, and screen-printing methods. These fillers rely solely on noncovalent interactions (van der Waals force and hydrogen bonding) to deposit on the MF skeleton, while such a noncovalent bonding approach may face filler shedding problems during the long-term service process. Besides, incorporating highly conductive fillers often significantly diminishes the thermal insulation property of foams. Therefore, the rational component and structural design of internal conductive network based on MF are necessary to solve the above issues.

Recently, the intrinsic conductive polymers represented by polypyrrole (PPy) have garnered significant interest attributed to their superior optical-electrical characteristics and environmental stability [20]. PPy is a polymer with strong shape plasticity, allowing for the manipulation of various properties through adjustments in the synthesis process. By altering the molar ratio of monomer/oxidant or monomer/initiator, and by selecting different types of oxidants or dopants, the nanostructure of PPy can be effectively controlled and fine-tuned [21, 22]. In a variety of nanostructures, PPy nanowire arrays are arranged neatly, which retain not only reversible doping/de-doping process and photothermal conversion property but also exhibit unique electric activity owing to the significant quantum effects, specific surface area, and size of nanomaterials [23]. The electrochemical polymerization method can be used to prepare PPy nanowire arrays with controllable size and orderly structure [24, 25]. The length and diameter of PPy nanowire arrays can be regulated by controlling monomer/electrolyte concentration, current density, polymerization time, and other parameters. The architectural design of micro-nano structures within macro-composites preserves the intrinsic chemical and physical traits of the constituent materials while ingeniously harnesses the unique structural configurations to achieve properties or specialized functionalities [26–28]. For example, Cheng et al. prepared PPy nanoarrays on hydrophilic carbon cloth via electrochemical polymerization, which was conductive to obtain multiple light absorption and served as an absorber to construct an interfacial solar evaporator [29]. Ning et al. fabricated porous membrane with PPy nanotip arrays via a surface-initiated polymerization, which showed an intelligent reversible superwettability by tailoring the redox states and height of PPy nanotips [30]. Multi-layer structure design of materials on the nano-micro scale is an efficient method to realize multifunctional integration. Therefore, the ordered PPy nanowire arrays constructed on the MF substrate have the advantages of nano-micro scale structure and high porosity, rendering the foams attractive options for EMI shielding and thermal insulation performances. However, to the best of the current research understanding, constructing PPy nanowire arrays on MF skeleton to enhance EMI shielding and infrared stealth performances has rarely been reported. The following challenges should be solved in this regard: (1) uniform growth of PPy nanowire arrays on MF substrate by electrochemical polymerization, (2) obtaining a hierarchical PPy foam which contained 3D porous PPy micro-skeleton and 1D PPy nanowire arrays, and (3) investigating the infrared stealth and EMI shielding mechanism of MF@PPy foams. Therefore, constructing ordered PPy nanowire arrays on MF substrate is highly desirable for fabricating multifunctional EMI shielding foams with hydrophobicity, thermal insulation, and infrared stealth functions.

**Fig. 1:**
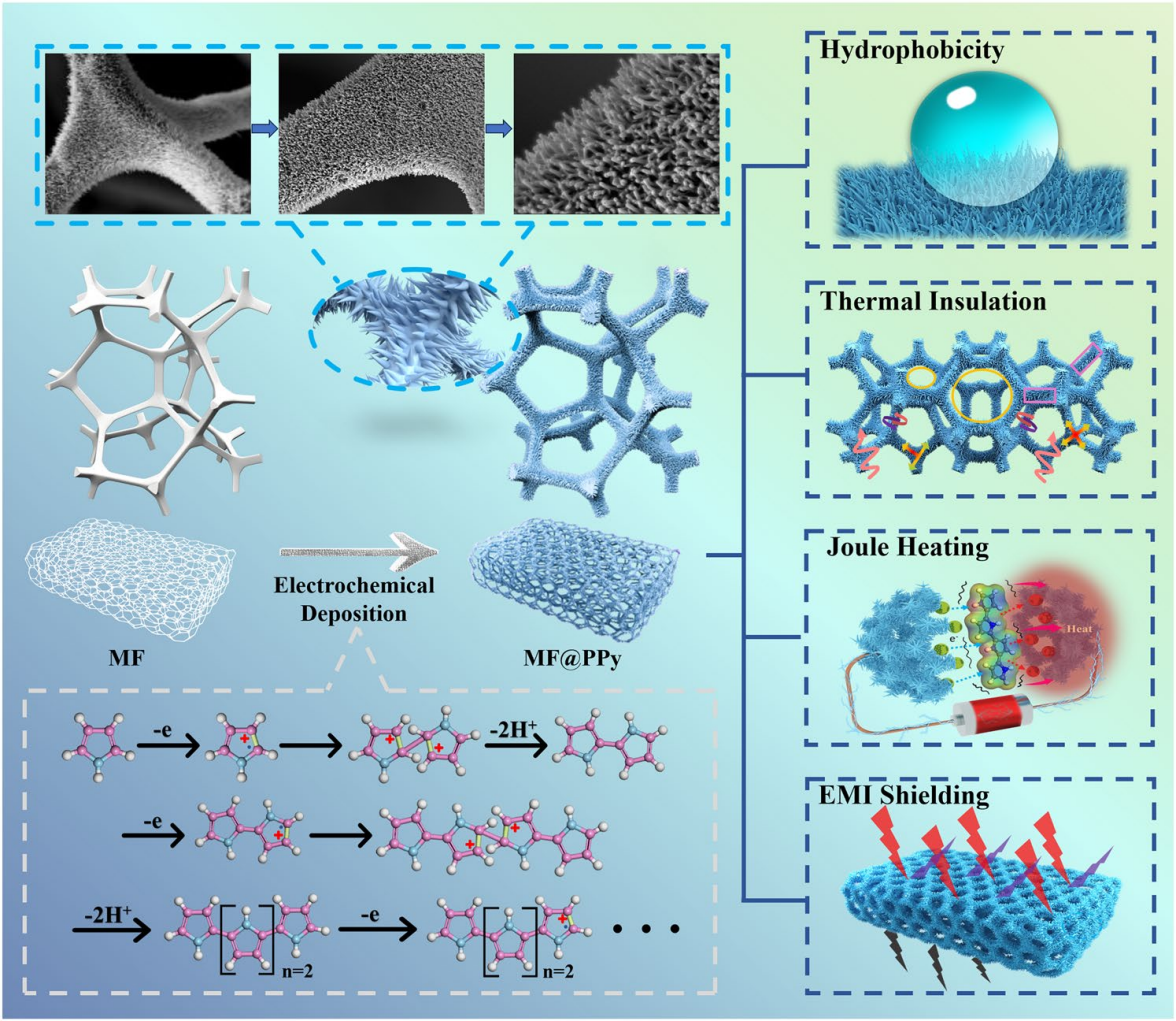
Schematic diagram showing the polymerization mechanism and multiple functions of MF@PPy foam. Atop well-ordered PPy nanowire arrays on the MF skeleton were grown by electrochemical polymerization. The hierarchical MF@PPy foam was composed of 3D PPy micro-skeleton and 1D PPy nanowire arrays. The MF@PPy foam achieved multifunctional integration including hydrophobicity, thermal insulation, dynamic infrared thermal camouflage and electromagnetic interference shielding properties

Herein, the directed growth of PPy nanowire arrays on the MF substrate through precise control of electrochemical polymerization conditions was successfully achieved, resulting in a unique three-dimensional and one-dimensional hierarchical structure. The hierarchical structure of the continuous PPy network along MF skeleton and upwardly grown PPy nanowire arrays achieved rapid electron transport channels, improved mechanical flexibility, and powerful multifunctional integration (Fig. 1). (1) Hydrophobicity: the PPy nanowire arrays increased the roughness surface of MF@PPy foams and enhanced the Cassie–Baxter state, resulting in high hydrophobicity. (2) Thermal insulation: the low thermal conductivity and elongated ligament characteristic of PPy nanowire arrays imparted the MF@PPy foams with excellent heat insulation ability. (3) Joule heating and infrared stealth: thanks to the superior electrical conductivity, the MF@PPy foams exhibited a rapid response to heating, reaching temperatures of up to 80 °C with just a 3 V voltage applied, leading to desirable Joule heating and active/positive infrared stealth performances. (4) EMI shielding performance: the hierarchical porous structure, as well as PPy nanowire arrays, enabled the MF@PPy foams to fully absorb electromagnetic waves through multiple attenuations, thus showing impressive EMI shielding effectiveness values of 55.77 dB and 19,928.57 dB cm^2^ g^−1^. The multifunctional MF@PPy foams can significantly enlarge the scope of EMI shielding applications, catering to both civilian and military requirements. This work proposes an innovative strategy for the fabrication of multifunctional EMI shielding foams via constructing PPy nanowire arrays, which can be applied on other porous materials.

## Experimental Section

### Materials

Pyrrole (Py) and p-Toluenesulfonic acid (p-TSA) were purchased from Shanghai Aladdin Biochemical Technology Co., Ltd. Sodium phosphate dibasic dodecahydrate (Na_2_HPO_4_·12H_2_O) and sodium dihydrogen phosphate (NaH_2_PO_4_) were purchased from Shanghai Maclean Biochemical Technology Co., Ltd. Anhydrous ethanol (EtOH) was purchased from Chengdu Chron Chemicals Co., Ltd. Melamine Foam (MF) was purchased from Anyin Acoustic Materials. The deionized (DI) water used was made in the laboratory.

### Pre-treatment of Py and MF

The Py was distilled to remove the inhibitor and oxidized Py in a vacuum distillation at 55 °C. The treated Py was stored in a N_2_ atmosphere at a low temperature in the dark. MF underwent ultrasonic cleaning processes with deionized water and EtOH to remove contaminants.

### Preparation of Electrolyte for PPy Synthesis

Initially, a mixture was prepared by combining 40 mL of 0.2 M NaH_2_PO_4_ and Na_2_HPO_4_, and the pH of this solution was adjusted to 6.66. Py and p-TSA were introduced into the solution in a stoichiometric ratio of 10:1. To ensure a uniform dispersion of components, the resulting electrolyte mixture was homogenized for 1 min using a vortex mixer.

### Preparation of MF@PPy Foams

The processed MF was cut into a fixed size (20 × 20 × 2 mm), and then, a 30-nm gold layer was deposited on the MF skeleton by magnetron ion sputtering instrument (GVC-2000, China), forming a circuit. PPy was synthesized through a galvanostatic deposition process, utilizing a current density of 0.6 mA cm^−2^. The reaction was executed under low-temperature conditions and in complete darkness to mitigate the risk of undesired self-polymerization of the Py monomer.

The MF@PPy*X* foams (where *X* corresponds to the reaction time) were prepared with specific 1, 2, and 3 h reactions, respectively. Each electrochemical synthesis sample was prepared using a freshly prepared electrolyte to ensure optimal conditions. Post-synthesis specimens were submerged in DI water, followed by multiple water changes to remove residual Py monomers and electrolytes. Subsequently, the samples were subjected to freeze-drying to remove the absorbed water, thereby preserving the original morphologies of the synthesized materials. For the electrochemical setup, the Ag/AgCl electrode served as the reference electrode, while a platinum mesh electrode functioned as the counter electrode in all experiments.

### Characterization

Field emission scanning electron microscope (FESEM, JSM 7800F, Japan), atomic force microscope (AFM, Multimode 8, USA), and optical microscope (LEICA DM 2700P, Germany) were used to observe the micro-morphologies of the MF and MF@PPy foams. Energy-dispersive X-ray spectroscopy (EDS, X-Max 80, UK) was adopted to analyze the elemental distribution in the MF@PPy foams. Fourier transform infrared spectroscope (FTIR, NIcolet iS 20, USA) and Raman spectra (Raman, LabRAM HR Evolution, Japan) excited using a 532-nm solid-state laser were employed to characterize the structural features of the MF and MF@PPy. X-ray photoelectron spectroscope (XPS, Thermo Scientific K-Alpha, USA) was further applied to demonstrate that PPy nanoarrays were successfully applied to the foam surface. Thermogravimetric analyzer (TGA, Netzsch 204F1, Germany) was employed to analyze the thermal stability of the MF and MF@PPy foams at a heating rate of 10 °C min^–1^ in a N_2_ atmosphere.

The water contact angles (WCAs) of the MF@PPy foams was measured by a drop shape analyzer (DSA25E, Germany). To characterize the thermal insulation performance of the MF@PPy, isotropic thermal conductivity was measured by a hot disk (TPS 2200, Sweden), and an infrared thermal imager (FLIR T620, USA) was used to record the heat transfer of the MF@PPy on the 45 °C hot platform. A Picoammeter (DAQ6510, USA) was applied to investigate the electrical conductivity ($\sigma$) of the MF@PPy foams (The electrical conductivity was calculated based on formula 1).

1 $$\sigma =\frac{L}{RS}$$

where $L$, $R$, and $S$ are the length, the resistance, and the cross-sectional area of the MF@PPy foams, respectively. Moreover, the electrical-thermal conversion of the MF@PPy foams was measured using a DC power source (RXN-603D, China) and an infrared thermal imager.

The EMI shielding effectiveness (SE) of the MF@PPy was evaluated from the $S$ parameters (including ${S}_{11}$, ${S}_{21}$) measured by a vector network analyzer (PNA E501C, China) in 8–12 GHz region (X-band), where the sample sizes used were 24 × 11 × 2 mm. The reflection coefficient ($R$), transmission coefficient ($T$), absorption coefficient (*A*), reflection SE (${SE}_{R}$), absorption SE (${SE}_{A}$), total SE (${SE}_{T}$), and shielding efficiency ($k$) could be calculated based on the following formulas:

2 $$R={|{S}_{11}|}^{2}$$

3 $$T={|{S}_{21}|}^{2}$$

4 $$A=1-R-T$$

5 $$S{E}_{R}=-10\mathit{log}(1-R)$$

6 $$S{E}_{A}=-10\mathit{log}(\frac{T}{1-R})$$

7 $$S{E}_{T}=S{E}_{A}+S{E}_{R}$$

8 $$k=100-\frac{1}{{10}^{S{E}_{T}/10}}\times 10$$

## Results and Discussion

### Morphology and Structure Characterization of MF@PPy Foams

**Fig. 2:**
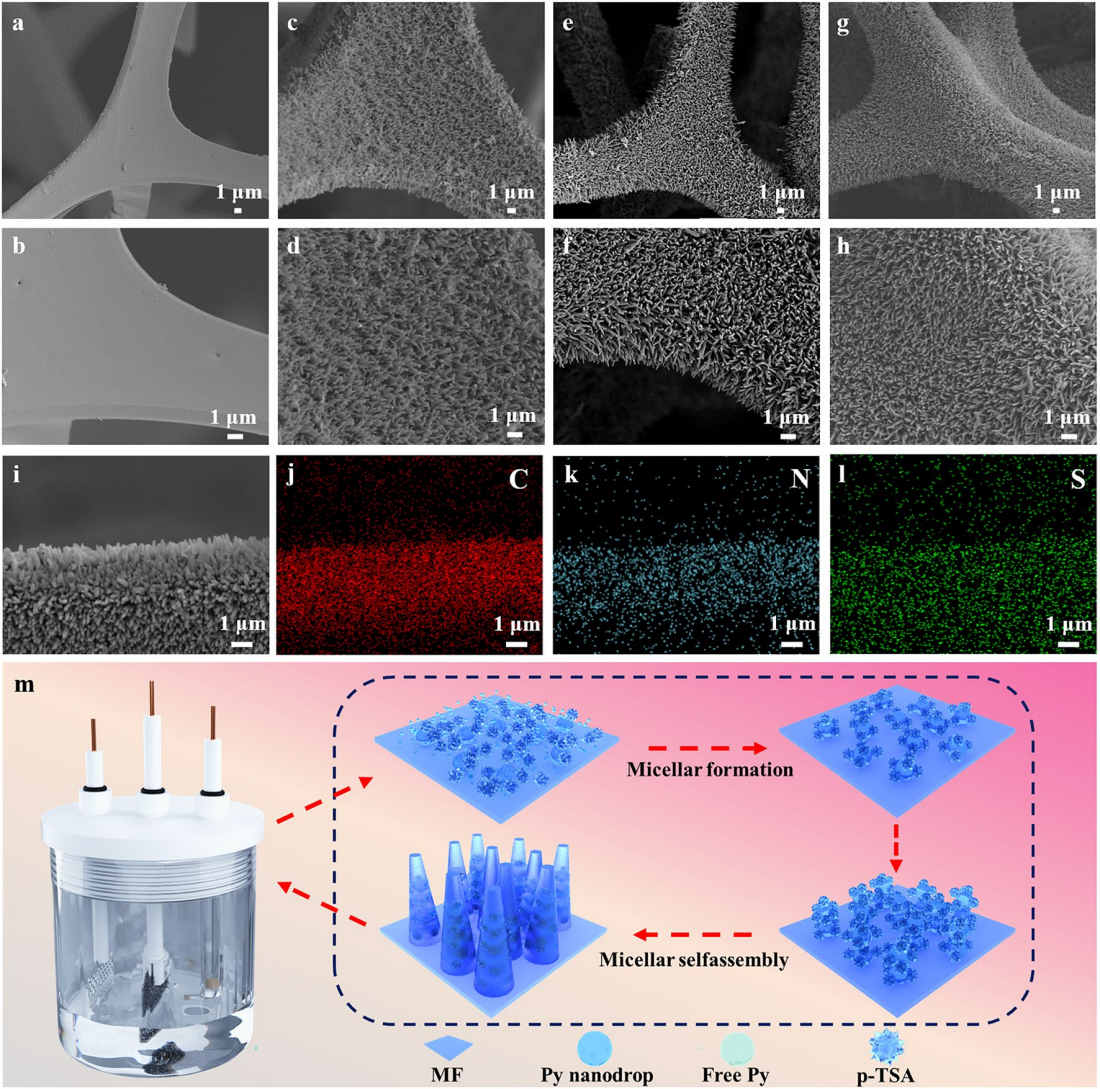
Microstructure of the MF and MF@PPy foams. FESEM images of **a, b** MF, **c, d** MF@PPy1, **e, f** MF@PPy2, and **g, h** MF@PPy3. **i-l** EDS analysis of the MF@PPy. **m** Schematic diagram of electrochemical polymerization of PPy nanowire arrays

Electrochemical synthesis stands out for its gentle experimental conditions and exceptional tunability, allowing for precise control over the structure of PPy nanowire arrays. Key parameters such as the electrolyte composition, reaction duration, solution temperature, and applied current can be finely adjusted to tailor the structural characteristics of PPy nanowire arrays [31, 32]. In this work, PPy nanowire arrays were prepared via one-step electropolymerization of Py on a uniform gold-sprayed MF by changing polymerization time (Fig. S1). As depicted in Fig. 2a and b, the pristine MF exhibits a uniformly smooth and flawless framework. The introduction of PPy nanowire arrays results in a noticeably roughened surface topography, indicative of the successful nucleation and growth of PPy nanowire arrays on the MF substrate. The 3D interconnected microstructure of MF still keeps intact after the polymerization of PPy, thus forming a multi-scale structure which consists of micrometer scale holes and nanoscale arrays. Notably, anchored PPy nanowire arrays show an extremely uniform morphology and a highly upright orientation. Meanwhile, PPy nanowire arrays become progressively longer, more regular and denser as the polymerization reaction time increases (Fig. 2c-h), which are advantageous for the construction of a hierarchically porous structure and a robust conductive network. The EDS mappings of the MF@PPy foam display the uniform distribution of C, N, and S elements, while S element is derived from the p-TSA (Fig. 2i-l). Figure 2m illustrates a schematic of the growth mechanism for PPy nanowire arrays, clarifying the step-by-step processes that contribute to the development of these ordered arrays. The Py monomers in the electrolyte exhibit two distinct forms: free Py molecules and Py nano-droplets. Firstly, the free Py molecules are preferentially adsorbed onto the electrode surface and captured by p-TSA to form a stable micelle. Aromatic rings of Py molecules and p-TSA with the *sp*^2^ carbon may form π–π interactions. Once the electrochemical polymerization is initiated, these adsorbed free-Py molecules on electrode rapidly polymerize to form a nascent and rough PPy layer, which serves as a nucleation site for the subsequent adsorption of the Py micelles. As p-TSA micelles adhere to the surfaces of the Py nano-droplets, they promote an organized arrangement of these droplets [33]. Furthermore, SEM images of the cross-sectional of the MF@PPy at various stages of polymerization were captured to more visually observe the morphological evolution that occurs throughout the electrochemical deposition process of PPy (Fig. S2). This directional assembly results in the creation of a well-structured nano-array with p-TSA playing a crucial role in directing the self-assembly of Py nano-droplets into well-ordered PPy nanowire arrays. Therefore, electroactive MF@PPy foams with a hierarchical structure are fabricated by one-step electropolymerization, which are composed of 3D continuous PPy skeleton and 1D PPy nanowire arrays.

**Fig. 3:**
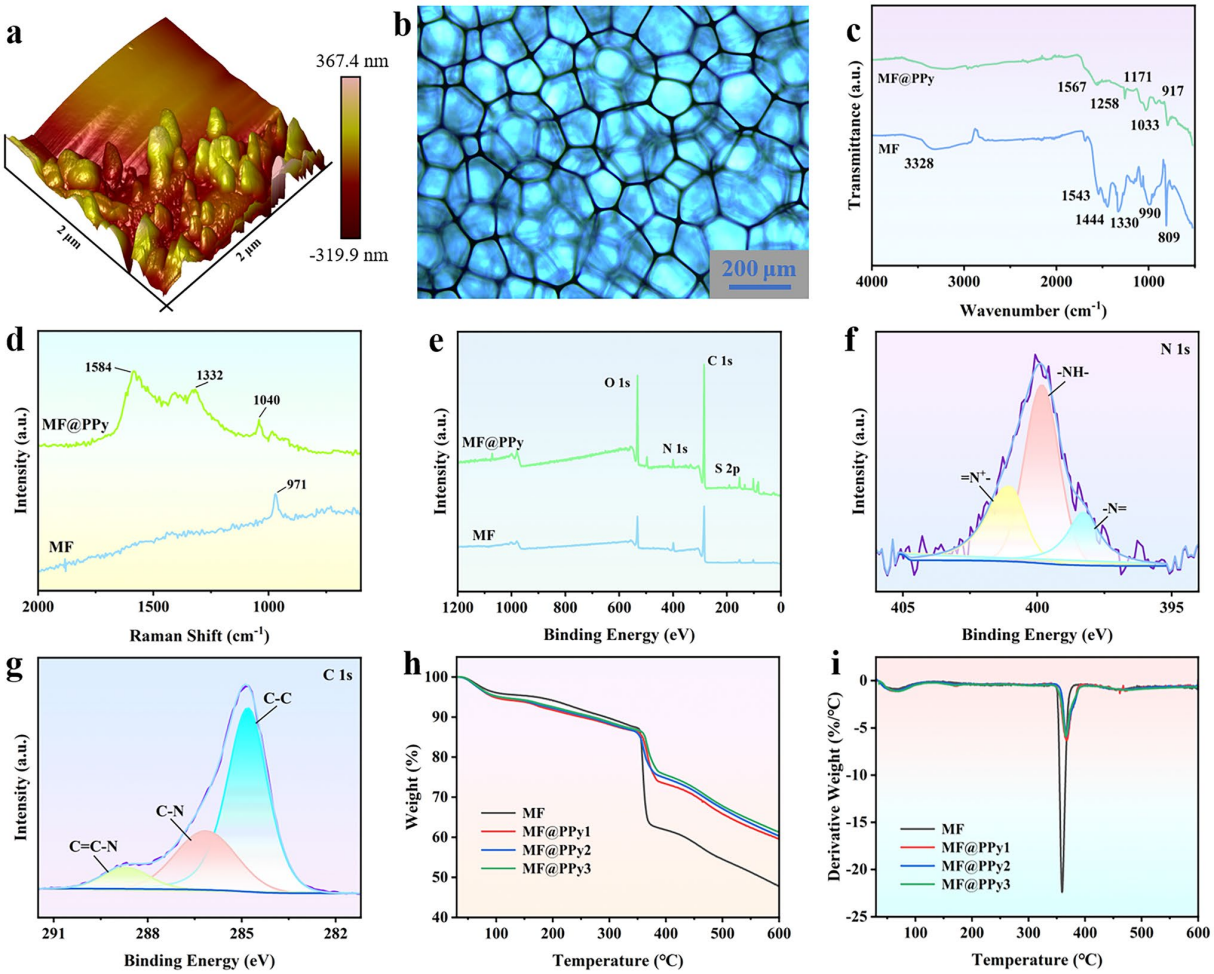
Structure characterization of MF@PPy foam. **a** AFM image of the MF@PPy foam. **b** Optical microscope image of the MF@PPy foam. **c** FTIR spectra of the MF and MF@PPy foams. **d** Raman spectra of the MF and MF@PPy foams. **e** XPS spectra of the MF and MF@PPy foams. **f** N 1*s* and **g** C 1*s* XPS spectra of the MF and MF@PPy foams. **h** TGA and **i** DTG curves of the MF and MF@PPy foams

The morphology of MF@PPy foams were further observed by AFM and POM measurements. The AFM image reveals that PPy nanowire arrays grew on the MF skeleton exhibit an increased surface roughness (Fig. 3a). Concurrently, the optical microscope shows that the MF@PPy retains its intact and continuous porous structure after electrochemical polymerization. The preservation of 3D skeleton confirms that the fundamental structural framework of the foam remains largely undisturbed (Fig. 3b). Figure 3c, d illustrates the FTIR and Raman spectral analysis of the pure MF and MF@PPy foams. The absorption peaks at 809, 1543, and 3328 cm^−1^ are assigned to the bending vibrations of the triazine ring, the stretching vibrations of the C = N bonds and the N–H bonds, respectively. Additionally, the peaks at 990, 1330, and 1444 cm^−1^ were corresponded to the stretching vibrations of the C–H bonds [34]. For MF@PPy foam, a distinctive new peak emerges at 1567 cm^−1^, which is indicative of the stretching vibration of the C = C bonds within the conjugated structure of the aromatic heterocycle present in PPy, and the peaks observed at 1258 and 1171 cm^−1^ are linked to the deforming vibrations of C–H bonds and stretching vibrations of C–N bonds. Additionally, the peaks appearing at 1033 and 917 cm^−1^ are ascribed to the stretching vibrations of C–C bonds and the out-of-plane vibrations of C–H bonds [35, 36]. Similarly, the Raman spectrum of the MF@PPy shows a new peak at 1040 cm^−1^ due to symmetrical C–H in-plane bending due to the introduction of PPy, which is different from the triazine ring nitrogen radial in-phase vibration at 971 cm^−1^ of pure MF [37]. Moreover, the peaks at 1332 and 1584 cm^−1^ peaks are associated with the antisymmetric C–N stretching and the C = C stretch vibrations of PPy [38, 39].

The chemical composition of MF@PPy was determined utilizing XPS measurements (Fig. 3e-g). The observed O, N, and C elements are originated from PPy nanowire arrays and MF. In the N 1*s* spectrum, three distinct peaks are observed at binding energies of 401.0, 399.8, and 398.3 eV. These peaks are associated with different nitrogen-containing structures within the PPy chains: = N^+^–, –NH–, and –N = . The high-resolution C 1*s* peaks at 288.7, 286.2, and 284.8 eV are assigned to C = C–N, C–N, and C–C in the PPy nanowire arrays, respectively [40, 41]. Figure 3h and i shows the TGA and derivative thermogravimetric (DTG) curves of pure MF and MF@PPy foams. The thermal decomposition process of MF and MF@PPy can be primarily demarcated into two distinct phases. During the initial phase (30–120 °C), a weight reduction of approximately 6% is recorded. This mass loss is ascribed to the evaporation of moisture adsorbed within the structure of the materials. The second phase (333–395 °C) is characterized by a more significant mass loss, primarily ascribed to the thermal scission of chemical bonds within the MF skeleton and the PPy nanowire arrays [40, 42, 43]. Furthermore, the TGA curves reveal an interesting trend in the residual mass of the MF@PPy foams. It is observed that the residual mass of the MF@PPy foams increases as the polymerization time of PPy increases. This increase in residual mass is due to high concentration of PPy nanowire arrays, which are known for their ability to leave behind a more substantial amount of residual carbon upon thermal decomposition. Concurrently, the DTG curves indicated a shift in the peak of the weight loss rate to higher temperatures, suggesting that the introduction of PPy contributes to a heightened thermal stability. This enhancement is likely due to the inherent high thermal stability of PPy and the heterocyclic structure of PPy, which is doped with p-TSA [44]. Based on the above results, the upwardly grown PPy nanowire arrays are successfully grown on the MF skeleton, which are critical for excellent integrative properties.

### Hydrophobicity of MF@PPy Foams

**Fig. 4:**
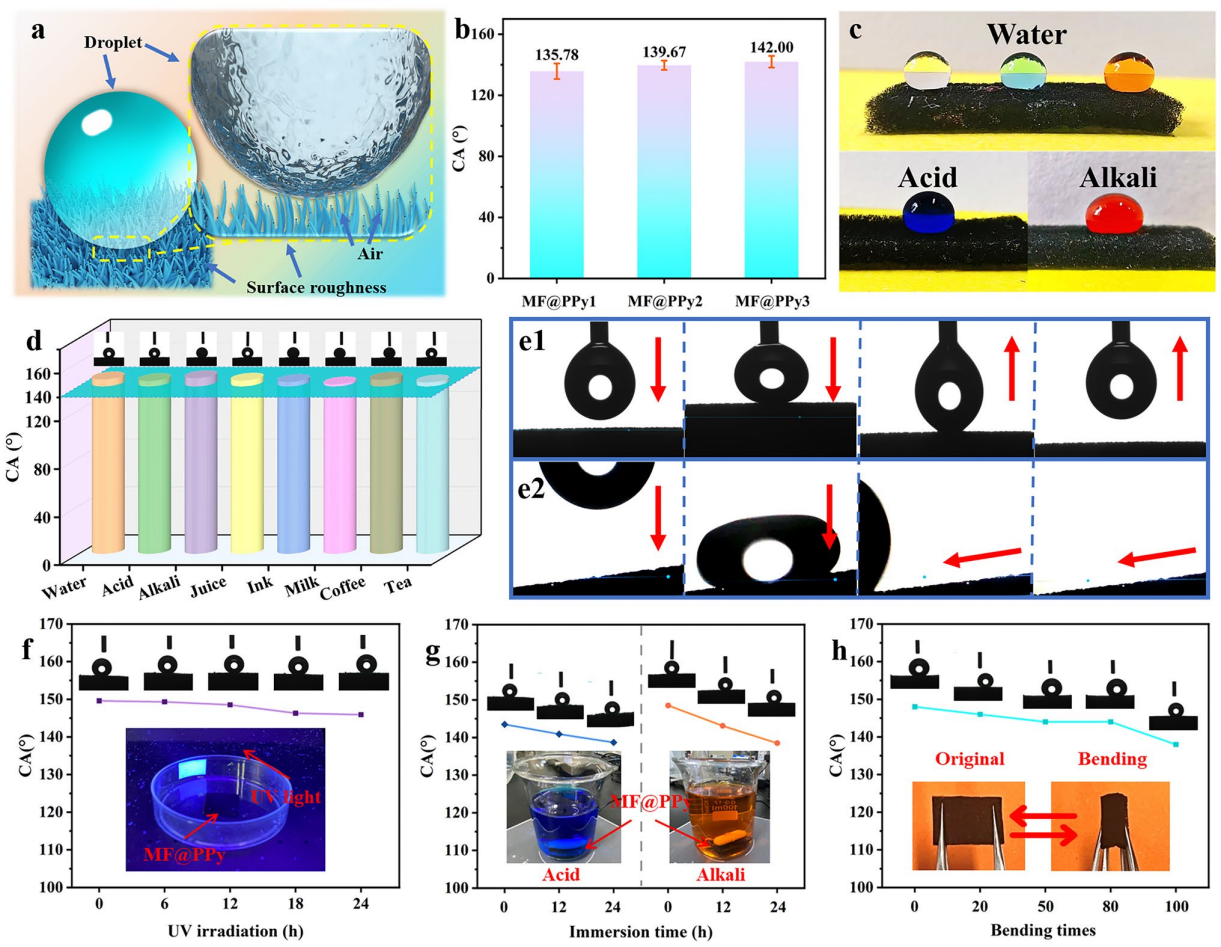
Hydrophobic property evaluation of MF@PPy foams. **a** Schematic diagram of the hydrophobic mechanism of MF@PPy. **b** The WCAs of MF@PPy1, MF@PPy2, and MF@PPy3. **c** Digital images of various liquid droplets on the MF@PPy surface. **d** The WCAs of various liquid droplets on the MF@PPy surface. **e1, e2** Diagram of a water droplet touching and rolling on the MF@PPy surface. The WCAs of MF@PPy after **f **UV irradiation, **g** chemical corrosion, and **h** multiple bending

Generally, achieving hydrophobic properties typically involves the integration of material with low surface energy along with the micro/nanoscale surface roughness [45, 46]. As mentioned above, PPy nanowire arrays with tunable length are obtained by controlling the growth time. The geometric structure of PPy nanowire arrays may play a pivotal role in the water wettability of the hierarchical MF@PPy foams. In order to enhance the practical applicability, a dip-coating technique was employed to apply a hydrophobic polydimethylsiloxane (PDMS) coating onto the MF@PPy surface. Given the dilute concentration of PDMS at a mere 1wt‰, its introduction into the system had a negligible impact on the morphology of the PPy nanowire arrays. This ratio ensured that the intrinsic structure and texture of the PPy nanowire arrays were preserved, maintaining their pristine form and function (Fig. S3). The micro-nano dual-scale roughness surface of MF@PPy foams provides the necessary topographical complexity that enhances the Cassie–Baxter state [47, 48], where air pockets are trapped beneath the water droplets, thereby significantly reducing the contact area with the MF@PPy foams and enabling the self-cleaning effect (Fig. 4a). As shown in Fig. 4b, the WCAs of MF@PPy1, MF@PPy2, and MF@PPy3 are 135.78°, 139.67°, and 142.00°, respectively. Herein, PPy nanowire arrays gradually grow as the polymerization time increases, enhancing the prominence of the micro-nano structure and thus obtaining high hydrophobic property. The superior hydrophobic property of MF@PPy imparts these foams with a robust resistance to water, acid, and alkali corrosive agents (Fig. 4c). Due to its high hydrophilicity, the pristine MF is susceptible to contamination in practical applications and may struggle to withstand harsh environmental conditions (Fig. S4). In contrast, water droplets are capable to remain stable atop surface the surface of the MF@PPy foams without penetrating them. The acid and alkali solutions can keep still when coming into contact with the foam surface, which is indicative of commendable corrosion resistance. Furthermore, Fig. 4d shows the wettability of the MF@PPy foams to common liquids, the WCAs of all droplets are higher than 140°, and it is evident that the droplets assume a near-spherical form on the surface of foam, distinctly resting atop rather than seeping into the foam. Figure 4e1 and e2 presents a vivid illustration of the hydrophobic property of MF@PPy foam. The foam was moved upward until it contacted a 4 μL DI water droplet that was suspended in the air, the droplet’s shape was transformed from a circle to an ellipse due to the gentle pressure applied at the point of contact. As the foam was subsequently moved downward, the water droplet experienced stretching and deformation influenced by the adhesive forces at play on its interaction with the surface of the foam. This continued until the droplet eventually detached from the foam (Fig. 4e1). An 8 μL DI water was dropped vertically onto the MF@PPy foam with a slight inclination angle. The droplet hit the surface of the foam and deformed under gravity, then quickly rebounded to the original shape and promptly rolled off the foam (Fig. 4e2).

In addition, composite materials are frequently used in challenging and harsh environments, where they may inflict various external damage. Consequently, the durability and longevity of the hydrophobic properties of the MF@PPy foams are paramount. Figure 4f-h shows the WCAs of the MF@PPy foams after ultraviolet (UV) light, chemical corrosion, and bending, respectively. The UV resistance of foam was assessed through exposure to a UV lamp, during which the WCA was closely monitored for any alterations (Fig. 4f). It was observed that the WCA displayed only a slight change. Remarkably, after a continuous 24 h period of UV irradiation, the WCA of the MF@PPy3 was measured at 145.92°, demonstrating its enduring hydrophobic property even under prolonged exposure to UV light. In order to evaluate the acid and alkali resistance of the MF@PPy3, a durability test was conducted by immersing the foams in solutions with extreme pH levels: acidic solution (pH = 2) and alkaline solution (pH = 10) (Fig. 4g). Post a 24 h immersion period, the WCAs of the foam could still reach 138.70° and 138.50°, respectively, which proved that the MF@PPy3 possessed good acid and alkali corrosion resistance. Given the requirement for MF@PPy3 to endure bending in practical applications, maintaining the hydrophobicity even under such deformation is crucial. As shown in Fig. 4h, the MF@PPy3 maintained relatively good hydrophobicity ability after 100 cycles of bending, indicating their robustness against the deleterious effects of mechanical deformation. In addition, the hydrophobicity of the MF@PPy foams was tested after subjecting it to a 10 g weight and mechanical sliding on sandpaper (Fig. S5 and Movie S1). Hydrophobicity tests showed that the water contact angle of MF@PPy foams was maintained at an impressive 145.3°, which highlights the robust adhesion of the PPy nanowire arrays and the remarkable adaptability of the MF@PPy foams to complex and demanding working environments. This result demonstrates the durability and stability of our material, ensuring its effectiveness even under harsh conditions.

### Thermal Insulation Performance of MF@PPy Foams

**Fig. 5:**
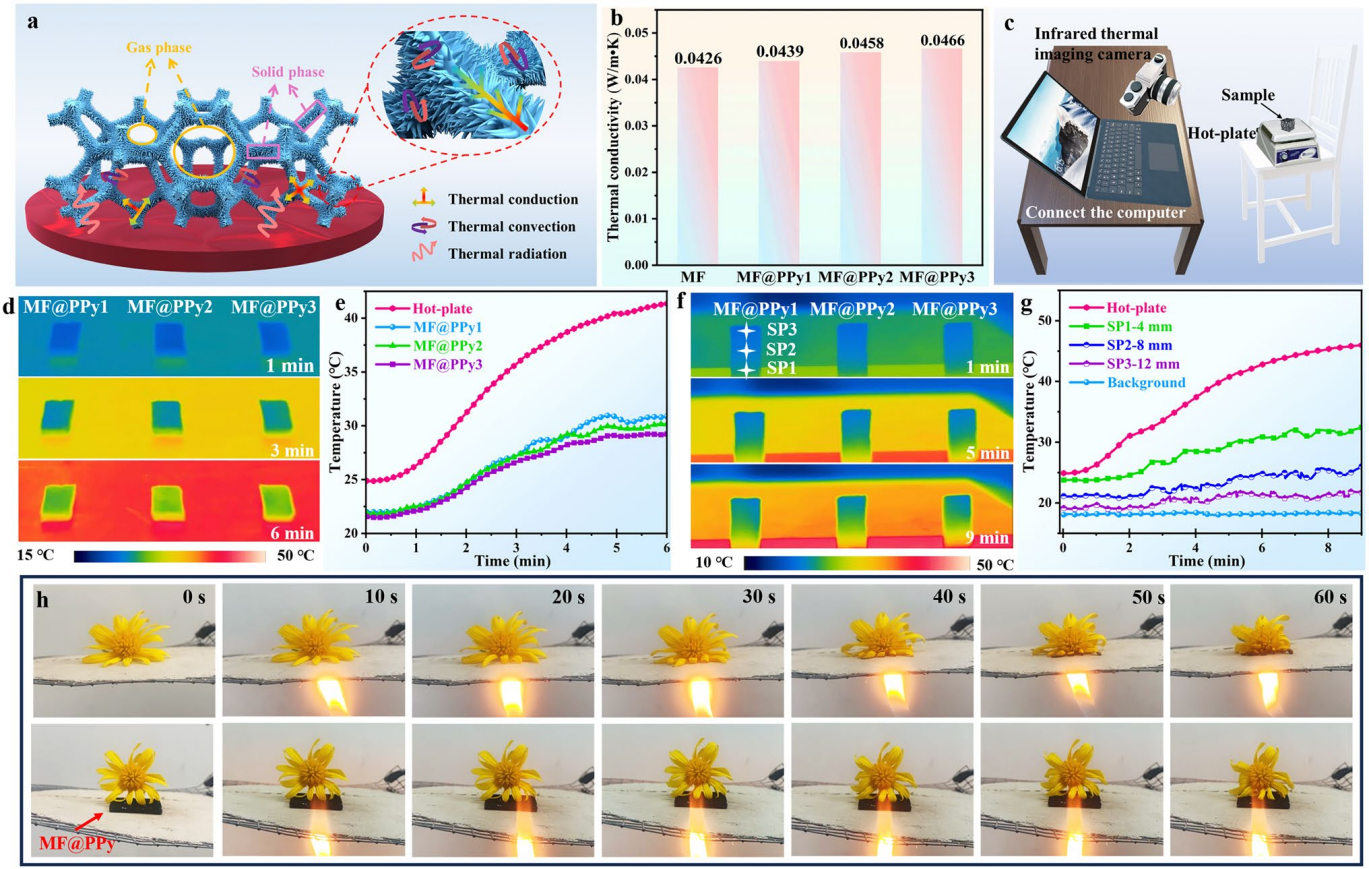
Thermal insulation performance of MF@PPy foams. **a** Schematic diagram showing the thermal insulation mechanism of MF@PPy foams. **b** The thermal conductivity of MF@PPy1, MF@PPy2, and MF@PPy3. **c** Schematic diagram showing infrared stealth performance test. Infrared thermal images and temperature curves of **d, e** MF@PPy foams placed parallelly on the hot stage and **f, g** MF@PPy foams stood upright on the hot stage. **h** Photographs of the flower heated by fire under the asbestos mesh and MF@PPy foam

Benefiting from the porous and hierarchical structure, the MF@PPy foams not only possess exceptional lightweight characteristics (0.014 g cm^−3^) but also serve as effective thermal insulators. The thermal insulation efficacy of the MF@PPy foams is illustrated in Fig. 5a. Heat transfer within these porous materials occurs through three principal mechanisms: conduction through the solid matrix, convection of gas within the structure of the foam, and radiation across the surface of the MF@PPy [14, 19]. The low density and high porosity of the MF@PPy foams create an extensive network of air pockets within their structure. The air trapped within these pockets possesses an inherently low thermal conductivity, which plays a dual role in enhancing insulating capabilities the MF@PPy foams. Firstly, it significantly diminishes the intensity of heat transfer through the solid phase, as the air pockets disrupt the continuous path that heat would otherwise follow through the material. Secondly, the presence of abundant air also reduces heat transfer by radiation, as the air pockets act as effective barriers to radiative heat exchange. Moreover, the low density of MF@PPy helps to reduce solid-phase heat conduction, since less material means less medium for heat to travel through. Additionally, the elongated ligaments characteristic of MF framework and PPy nanowire arrays can increase the tortuosity of thermal transfer path, effectively lowering the thermal conductivity by impeding the direct passage of thermal energy. Figure 5b shows the thermal conductivity of MF@PPy foams, and pure MF has an inherently low thermal conductivity, which is quantified at only 0.0426 W m^−1^ K^−1^. Extending the polymerization time results in a slight increase in the heat transfer rate for MF@PPy foams. Specifically, MF@PPy3 exhibits a thermal conductivity of 0.0466 W m^−1^ K^−1^. Despite this increase, the value remains in the low thermal conductivity range, confirming its use as a thermal insulating material. To conduct a direct assessment of the thermal insulation capabilities of the MF@PPy foams, an infrared thermal imager was deployed to observe the temperature variations across the foams on a heating platform (Fig. 5c). The MF@PPy foams were positioned on a heating platform with the temperature raised from room temperature to 45 °C. The infrared thermal imaging data revealed that even after a duration of 6 min of exposure to heat, the surface of MF@PPy foams persistently exhibited a lower temperature level (Fig. 5d). During this process, the temperature of the MF@PPy foams incrementally rose to approximately 30 °C and then was tended to be stable (Fig. 5e). Moreover, the MF@PPy foams exhibited a slower heating rate compared to the heating platform due to the passive thermal insulation capability. In addition, the MF@PPy foams exhibited outstanding thermal insulation properties even at elevated temperatures. Remarkably, the foams were capable of reducing the temperature to nearly 85 °C on a 150 °C heating platform, which was a significant cooling (Fig. S6). Figure 5f, g demonstrates further exploration of the thermal insulation characteristics of the MF@PPy foams with varying thicknesses. The infrared thermal images provide a visual representation of the temperature distribution across the MF@PPy foams. Notably, the upper and middle regions of the foams exhibited thermal signatures that closely matched the ambient environment, with only subtle variations observed in the lower section. The temperature profiling data corroborated this observation, indicating that while the bottom layer reached the highest temperature, it remained within a moderate range, peaking at around 30 °C. Conversely, the upper and middle regions demonstrated an exceptional ability to sustain temperatures equivalent to the surrounding environment over an extended period. By inhibiting the transfer of heat, the MF@PPy foams maintained a surface temperature that did not rise dramatically. This resulted in a lower overall temperature, effectively providing thermal camouflage for hot objects. Importantly, Stefan–Boltzmann’law dictates that the thermal radiation emitted by an object is directly proportional to its surface temperature and its infrared emissivity [49, 50]. The emissivity of a surface, which quantifies its effectiveness in emitting thermal radiation relative to a black body at the same temperature, is a crucial parameter for reducing the detectability of objects in infrared detection systems. Herein, MF@PPy foam exhibits an average emissivity of 0.55 (Fig. S7). This low emissivity diminishes the intensity of the thermal radiation emitted from the surface of the object. Consequently, the radiant temperature recorded by thermal imaging cameras is reduced, making the object less visible to infrared detection systems. The combination of the superior thermal insulation property and inherently low infrared emissivity endows MF@PPy foams with exceptional infrared stealth characteristics. This dual advantage effectively lowers the thermal signature, thereby diminishing detectability to infrared surveillance equipment.

As shown in Fig. 5h, when the flower was placed on the asbestos mesh and heated continuously by fire, it was observed that the flower could not tolerate the heat and collapsed due to wilting. In contrast, the introduction of MF@PPy foam beneath the flowers as a protective layer under identical heating conditions yielded markedly different phenomenon. The flower maintained its structural integrity, mainly due to the effective thermal insulation provided by the MF@PPy foam. Furthermore, the MF@PPy foam served a dual function as a thermal shield, effectively minimizing heat transfer to the flower (Fig. S8 and Movie S2). It also exhibited commendable flame retardant properties, preserving its structural integrity even when subjected to the intense heat of a flame (Movie S3). The excellent thermal insulation and flame retardant capabilities of MF@PPy foams make them suitable for high-temperature applications.

### Electric-Thermal Conversion Performance of MF@PPy Foams

**Fig. 6:**
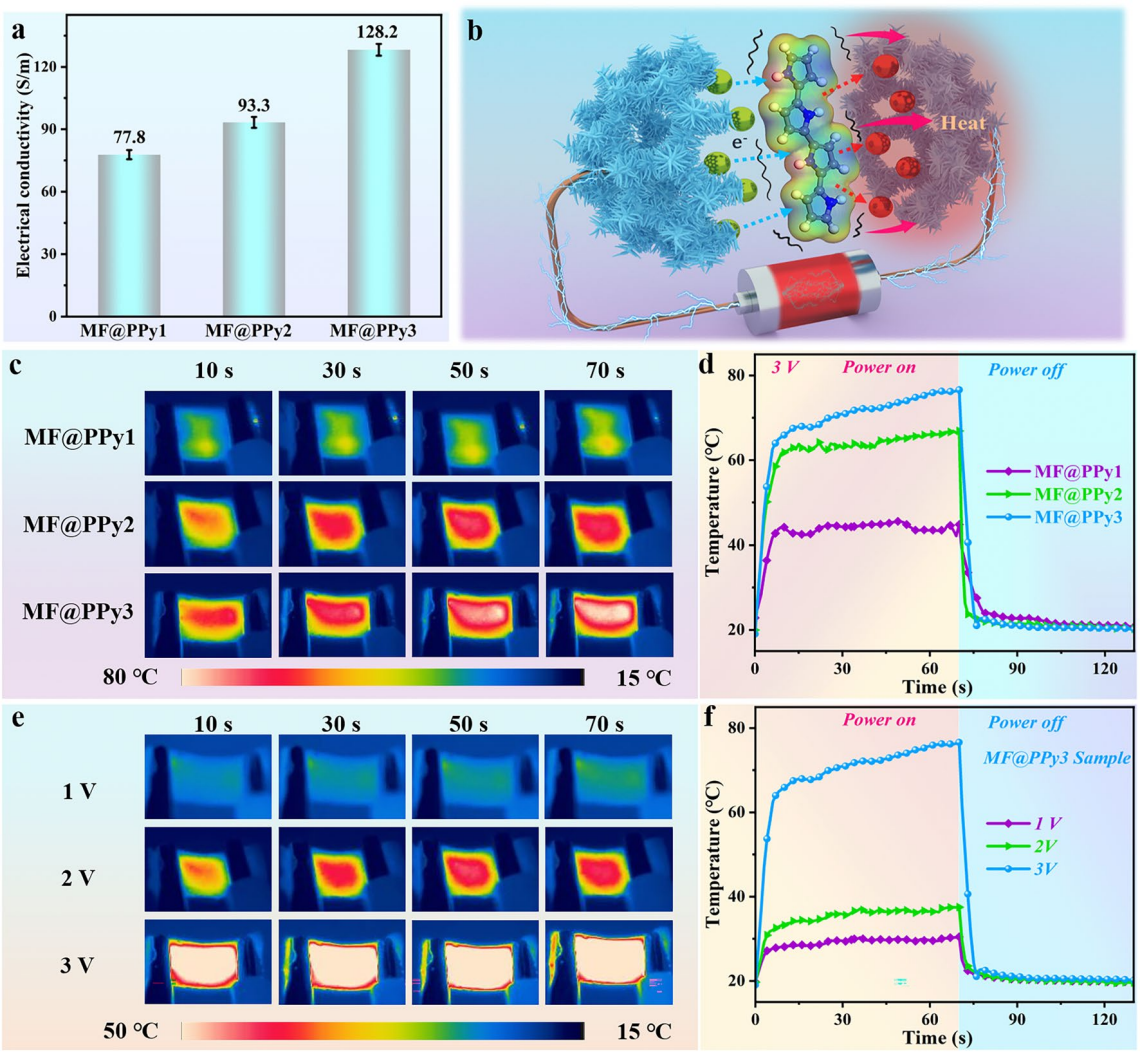
Electric-thermal conversion performance of MF@PPy foams. **a** Electrical conductivity of MF@PPy foams. **b** Schematic diagram of electric-thermal conversion mechanism of MF@PPy foams. **c** Infrared thermal images and **d** temperature–time curves of MF@PPy foams at 3 V voltage. **e** Infrared thermal images and **f** temperature–time curves of MF@PPy3 at different voltages

Traditionally, thermal camouflage materials rely on passive infrared stealth techniques, which primarily involve delaying the rate of temperature increase through their inherent thermal insulation properties or utilizing low infrared emissivity coatings. Furthermore, incorporating electric-thermal conversion technology into thermal camouflage materials significantly enhances their adaptability. This innovative approach allows for the precise control of surface temperature, enabling the generation of a deceptive heat signature through active Joule heating to mislead infrared detection systems. In this work, electrically conductive MF@PPy foams contained PPy nanowire arrays are obtained after doping with p-TSA. As depicted in Fig. 6a, there is a discernible trend that increasing electrical conductivity with extended polymerization time. Notably, the electrical conductivity of the MF@PPy3 foam reaches an impressive value of 128.2 S m^−1^. This enhancement is potentially due to the progressive refinement of the conductive pathways as the polymerization time increases, enabling more efficient movement of π-bonded electrons. Figure 6b shows the mechanism of electric-thermal conversion of the MF@PPy foams. Under the influence of the applied voltage, electrons inside the MF@PPy foams move along the conductive pathways, generating an electric current. As the electrons migrate, they collide with other molecules or groups to generate Joule heating, thus facilitating the process of electric-thermal conversion [51]. Infrared thermal images showed that the surface temperature of the MF@PPy foams rapidly ascended due to Joule heating when subjected to a 3 V supply voltage and quickly reached a steady-state temperature (Fig. 6c). The time–temperature curve shown in Fig. 6d indicated that the MF@PPy3 foam reached the highest temperature of around 80 °C, attributed to its superior electrical conductivity that allowed for increased transmission current, thereby enhancing the Joule heating effect. As soon as the applied voltage was terminated, the temperature of the MF@PPy foams plummeted rapidly, indicating an impressive cooling rate. Furthermore, the temperature response of MF@PPy3 under varying voltages was experimentally investigated. The surface temperature of MF@PPy3 increased gradually and the heating rate was faster as the voltage increased. Even with PDMS impregnation, the MF@PPy foams exhibited stable and effective Joule heating capabilities. This indicated that the incorporation of PDMS did not significantly compromise the ability of MF@PPy foams to generate heat through electrical resistance (Fig. S9). Furthermore, the durability of the electric-thermal conversion performance of MF@PPy the foams was tested (Fig. S10). The maximum temperature remained nearly unchanged after multiple cycles of charging and discharging, and the response time to temperature changes was consistently rapid. These results highlight the robustness and reliability of the MF@PPy foams in maintaining Joule heating efficiency over time. Moreover, the maximum voltage applied during the experiments was only 3 V, which was far lower than the maximum value that the human body can withstand (36 V), thus adhering to energy-saving and safety standards. The excellent Joule heating performance at low voltage enables the MF@PPy foams to achieve low-energy electric-thermal conversion, and the wide temperature range also provides a large scope for thermal camouflage applications.

### Dynamic Infrared Stealth Performance of MF@PPy Foams

**Fig. 7:**
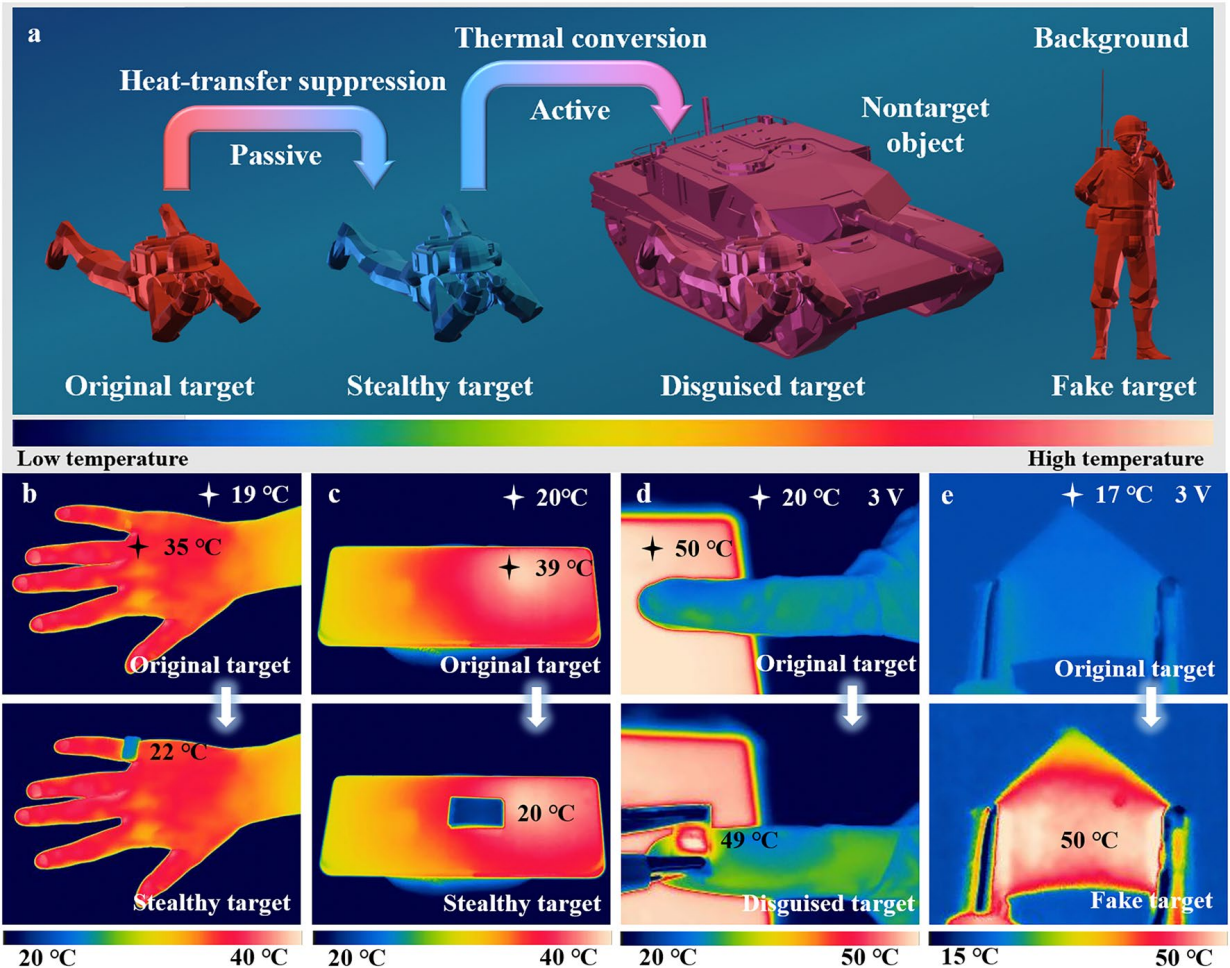
Infrared thermal camouflage application of the MF@PPy foams. **a** Schematic diagrams showing the active and passive thermal camouflage of MF@PPy foams in different environmental conditions. The infrared thermal camouflage of MF@PPy foam was applied to **b** the human body, **c** electronic devices, **d** low-temperature objects, and **e** false targets

To elucidate the infrared stealth or thermal camouflage capability of the flexible porous MF@PPy foams, Fig. 7a illustrates schematic diagrams of their application in various environmental conditions. In low-temperature settings, the MF@PPy foams are deployed as insulating layers over a high-temperature target, effectively utilizing their thermal insulation property to conceal the target’s heat signature. This passive approach to infrared stealth allows the target to blend seamlessly with the surrounding colder environment. Conversely, in high-temperature environments, the MF@PPy foams are applied to a low-temperature object. The foams are actively heated to synchronize with the ambient temperature by utilizing electric-thermal conversion, thereby achieving thermal camouflage for the cooler object. This process effectively disguises the object, rendering it inconspicuous against the warmer background. Moreover, the flexibility of MF@PPy foams allows them to be molded into specific shapes, and when combined with the application of an electric voltage, the foams can be used to create false heat signatures. This capability to generate deceptive thermal profiles can be strategically utilized to mislead adversaries, enhancing the utility in stealth and deception tactics (Fig. S11).

Figure 7b-e showcases the practical applications of the MF@PPy foams in infrared thermal camouflage. In a low-temperature environment of 19 °C, the MF@PPy foam was covered on the finger. Owing to the superior thermal insulation property of MF@PPy, the infrared thermal images revealed that the area covered by MF@PPy closely matched the ambient temperature. This similarity was visually represented by the uniform blue hue in the thermal image, indicating a successful concealment of the heat signature of the finger against the cold background (Fig. 7b). This phenomenon was further exemplified with a mobile phone, where the segment covered with MF@PPy showed a coloration akin to that of the surrounding environment, again represented by blue. In contrast, the uncovered portions of the mobile phone appeared in the medium to high-temperature range, as indicated by a distinct red color. This comparative contrast vividly illustrated the ability of MF@PPy to obscure the thermal signature of objects, effectively rendering them undetectable in infrared thermal imaging systems (Fig. 7c). Furthermore, the MF@PPy foams were also utilized to demonstrate thermal camouflage under high-temperature conditions. The MF@PPy foams were capable to be heated to match the ambient temperature by employing electric-thermal conversion, effectively concealing low-temperature objects within this high-temperature environment. In another instance, the MF@PPy was shaped to fit an electronic device. Through the same electric-thermal conversion process, it was heated to emulate the medium to high temperature characteristic of operational electronic devices (Fig. 7d, e). This active/positive manipulation of the thermal signal is crucial for achieving effective thermal camouflage, as it can deceive infrared detection systems by presenting misleading thermal information. Therefore, the strategic combination of thermal insulation and electric-thermal conversion dramatically enhances the functionality and effectiveness of the MF@PPy foams in various applications, such as military camouflage, surveillance technology, and other fields where concealment is essential.

### EMI Shielding Performance of MF@PPy Foams

**Fig. 8:**
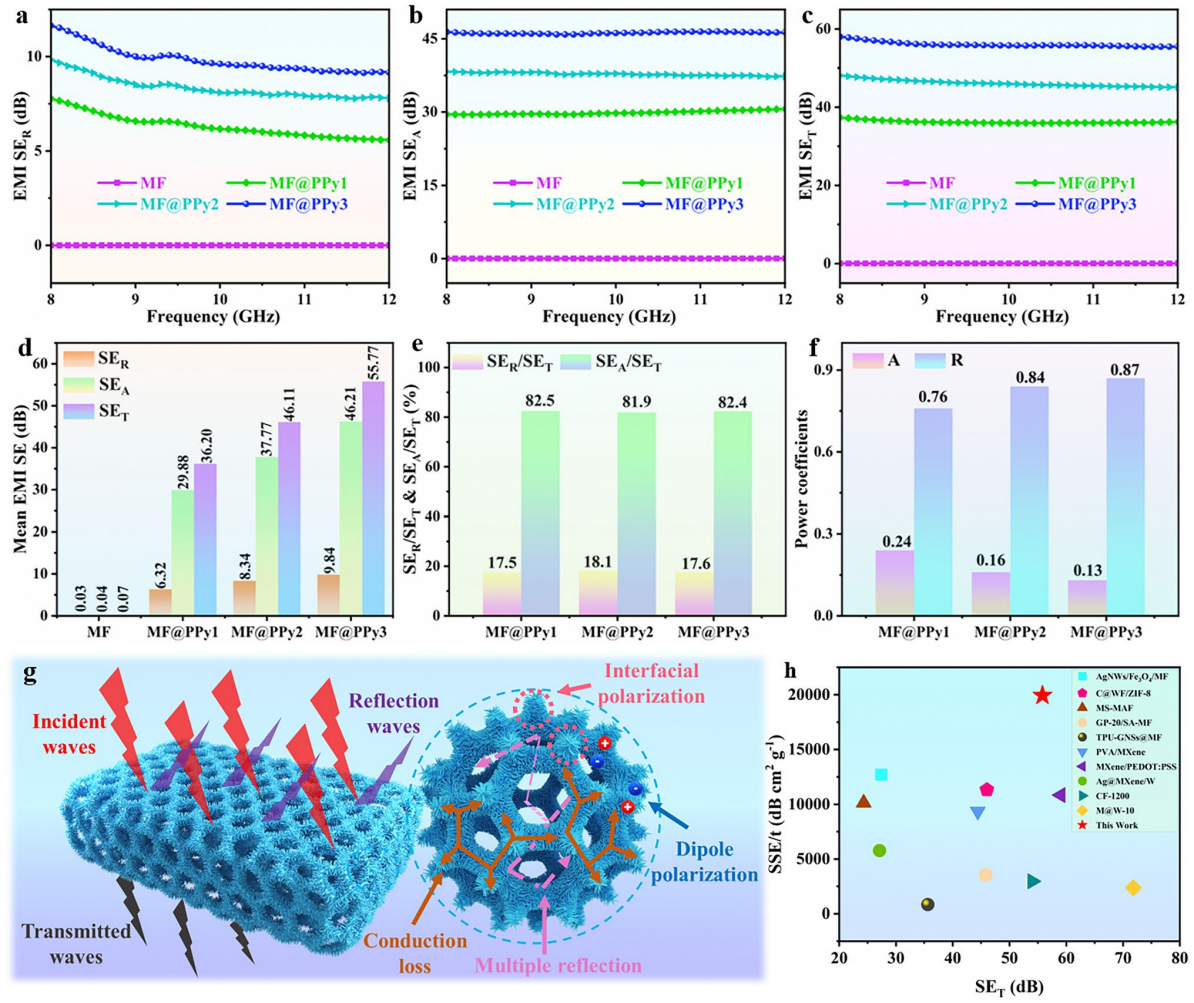
EMI shielding performances of MF@PPy foams. **a** ${SE}_{A}$, **b** ${SE}_{R}$, **c** ${SE}_{T}$, and **d** the mean $SE$ values of MF and MF@PPy foams. **e** ${SE}_{R}$/${SE}_{T}$ and ${SE}_{A}$/${SE}_{T}$ values of the MF@PPy foams. **f** Power coefficients of the MF@PPy foams. **g** Schematic diagram showing the EMI shielding mechanism of MF@PPy foams. **h** $SSE/t$ and ${SE}_{T}$ of MF@PPy foams compared with related works

In today's technologically advanced society, the pervasive presence of EMI poses significant threats to electronic devices and human health. As a result, effective EMI shielding is essential, particularly for military electronic systems and individual safety. The MF@PPy porous foams demonstrate not only commendable electrical conductivity but also superior EMI shielding capability. Figure 8a-c presents a comparative analysis of ${SE}_{A}$, ${SE}_{R}$, and ${SE}_{T}$ values for pure MF and MF@PPy foams. It is observed that the pure MF lacks any significant EMI shielding performance, whereas the MF@PPy foams exhibit impressive EMI shielding efficiency. It is noteworthy that the ${SE}_{T}$, ${SE}_{R}$, and ${SE}_{A}$ values of the MF@PPy foams increase proportionally with the polymerization time of the PPy nanowire arrays. Figure 8d shows the mean $SE$ values for both the pure MF and the MF@PPy foams. The pure MF exhibits a ${SE}_{T}$ value of merely 0.07 dB, which does not provide any meaningful EMI shielding. Conversely, the MF@PPy3 demonstrates a remarkable ${SE}_{T}$ value of 55.77 dB, which equates to shielding efficiency of over 99.997%, significantly exceeding the performance levels of commercial benchmarks. Analysis of the ${SE}_{R}$/${SE}_{T}$ and ${SE}_{A}$/${SE}_{T}$ values of the MF@PPy foams reveals that the ${SE}_{A}$/${SE}_{T}$ values consistently exceed the ${SE}_{R}$/${SE}_{T}$ values (Fig. 8e), indicating that the entered EMWs could be trapped in the MF@PPy foams and further attenuated by microwave absorption caused by conduction loss and multiple microwave reflections. This phenomenon contributes to reducing the secondary reflection pollution caused by EMWs to a considerable extent. Nonetheless, it is essential to recognize that evaluating the EMI shielding mechanism solely based on the comparison of ${SE}_{R}$ and ${SE}_{A}$ values is insufficient since reflection loss and absorption loss cannot represent the actual levels of reflected and absorbed power. For a thorough comprehension of the EMI shielding behavior, it is crucial to consider the power coefficients of the foams, which include the $R$ and $A$. The values of $R$ and $A$ represent the ability of shielding materials reflect and absorb the incident EMWs, thereby revealing the actual EMW energy loss. The $R$ values of the MF@PPy foams are 0.76, 0.84, and 0.87 higher than the $A$ values, respectively, indicating that the predominant mechanism of EMI shielding in the MF@PPy foams relies on reflection. The MF@PPy foams also were tested in high temperature and high humidity environments to assess the durability and stability of MF@PPy foams (Fig. S12). Despite the harsh testing conditions, the EMI SE of the MF@PPy foams remained largely unaffected. This can be attributed to the unique micro-nano structure and exceptional chemical stability of the PPy nanowire arrays, which enabled MF@PPy foams to maintain effective shielding performance even in adverse environmental conditions.

The EMI shielding mechanism of the MF@PPy foams is predominantly stem from the surface characteristics of the foams and their internal micro-nano porous structure [52, 53]. When EMWs encounter the surface of the MF@PPy foams, a significant fraction of the incoming EMWs is reflected due to the abrupt impedance mismatch at the air-material interface. This initial reflection serves as the first line of defense against EMI. For those EMWs that manage to penetrate the MF@PPy foams, they are first met with dissipation through ohmic losses, which are facilitated by the conductive framework of MF@PPy foams. Within this framework, electrons traverse the conductive network through a process of migration and hopping. This conductive absorption of energy significantly reduces the intensity of the EMWs as traveling through the foam. Furthermore, polarization loss is a significant factor that should not be overlooked. Upon doping, PPy generates polaritons, which can be considered as intrinsic dipoles within the PPy structure. The dipole polarization that arises is a crucial element in enhancing the EMI shielding effectiveness of the MF@PPy foams. The substantial disparity in electrical conductivity between MF and PPy leads to pronounced polarization at their interface, which further augments the attenuation of electromagnetic waves. Moreover, the intricate micro-nano porous architecture of the MF@PPy foams significantly elongates the transmission path of the EMWs. This extended path increases opportunities for multiple reflections and scattering events within the foams. These interactions further attenuate the EMWs through cumulative energy loss, thus contributing to the overall SE of the MF@PPy foams (Fig. 8g) [54–57]. In the current age of miniaturization and portability for electronic devices, the considerations of material thickness and density have taken on heightened significance. To attain a profound and detailed comprehension of the EMI shielding capabilities of MF@PPy foams, the specific EMI-SE ($SSE/t=SE/density/thickness$), has been introduced and calculated. As depicted in Fig. 8h, the calculated $SSE/t$ value for the MF@PPy composite foam reaches an impressive 19,928.57 dB cm^2^ g^−1^. This value significantly outperforms the majority of other porous materials, highlighting the exceptional EMI shielding performance of the MF@PPy foams [16, 58–66]. The high $SSE/t$ value demonstrates a testament to the efficiency and effectiveness of the MF@PPy foams in providing robust shielding against EMI, making them a prime candidate for use in the compact and lightweight designs of modern electronic devices.

## Conclusions

In summary, we reported the electrochemical polymerization of well-ordered PPy nanowire arrays on the skeleton of MF to construct hierarchical MF@PPy foams. The hierarchical structure of the continuous PPy network along MF skeleton and upwardly grown PPy nanowire arrays achieved powerful multifunctional integration: the PPy nanowire arrays enhanced the surface roughness of MF, imparting high hydrophobicity with a WCA of 142.00° and excellent stability under complex environments. The elongated ligament characteristic of PPy nanowire arrays and low thermal conductivity showed a synergistic effect on the thermal insulation property of MF@PPy foams. Furthermore, the MF@PPy foams displayed good Joule heating performance and infrared stealth ability. The MF@PPy foams exhibited rapid heating/cooling response and the surface temperature could reach 80 °C at 3 V low applied voltage, leading to dynamic infrared camouflage capability. More importantly, benefiting from the well-ordered electroactive PPy nanowire arrays, the hierarchical MF@PPy foams exhibited EMI shielding effectiveness values of 55.77 dB and 19,928.57 dB cm^2^ g^−1^. Incorporating the benefits of hydrophobicity, thermal insulation, efficient Joule heating, adept infrared stealth, and robust EMI shielding, the hierarchical MF@PPy foams show great application potential in wearable electronics, military, and other high-tech fields.

## Supplementary Information

Below is the link to the electronic supplementary material.

Supplementary file1 (MP4 13932 kb)

Supplementary file2 (MP4 44144 kb)

Supplementary file3 (MP4 21274 kb)

Supplementary file4 (DOCX 7707 kb)
