# Supplementary material for: Hierarchically Porous Polypyrrole Foams Contained Ordered Polypyrrole Nanowire Arrays for Multifunctional Electromagnetic Interference Shielding and Dynamic Infrared Stealth
Source: Nanomicro Lett. 2024 Dec 26;17:97. doi: 10.1007/s40820-024-01588-x (PMC11671463; doi:10.1007/s40820-024-01588-x)
Supplement: Supplementary file 4 — Supplementary file4 (DOCX 7707 kb) [file 40820_2024_1588_MOESM4_ESM.docx]

Supporting Information for

**Hierarchically Porous Polypyrrole Foams Contained Ordered Polypyrrole Nanowire Arrays for Multifunctional Electromagnetic Interference Shielding and Dynamic Infrared Stealth**

Yu-long Liu^1^, Ting-yu Zhu^1^, Qin Wang^1^, Zi-jie Huang^1^, De-xiang Sun^1^, Jing-hui Yang^1^, Xiao-dong Qi^1,^*, Yong Wang^1,^*

^1^School of Chemistry, Key Laboratory of Advanced Technologies of Materials (Ministry of Education), Southwest Jiaotong University, Chengdu 610031, P. R. China

* Corresponding authors. E-mail: [qxdjianhu@163.com](mailto:qxdjianhu@163.com) (Xiao-dong Qi); [yongwang1976@swjtu.edu.cn](mailto:yongwang1976@swjtu.edu.cn) (Yong Wang)

ORCID: 0000-0003-0655-7507 (Yong Wang)

### Supplementary Figures


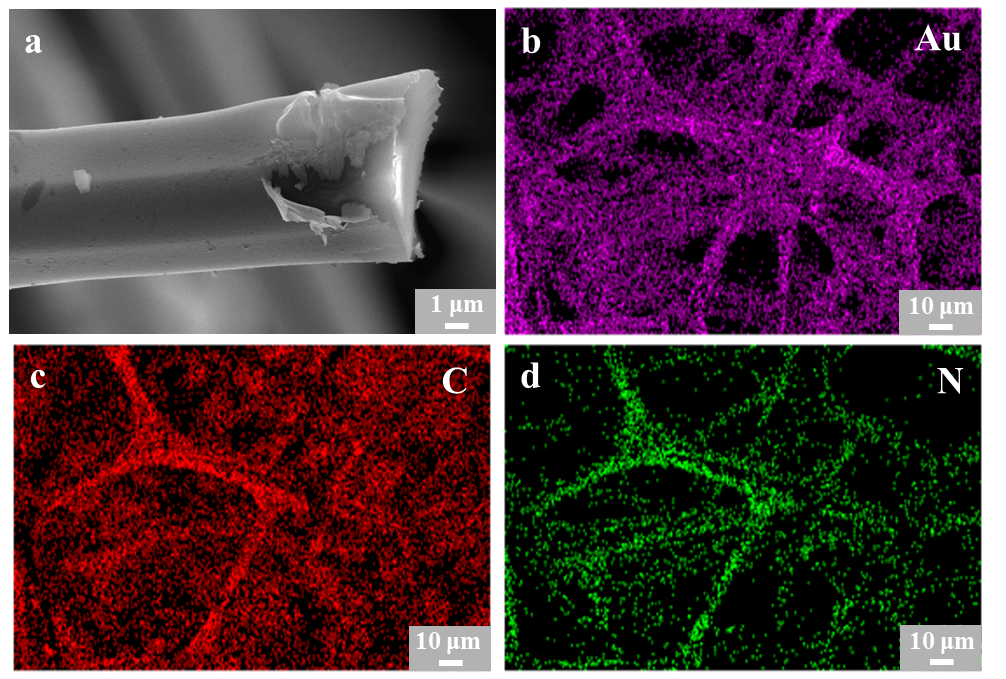


**Fig. S1** Low magnification SEM images and EDS of the MF (with uniform sputtered gold layer)


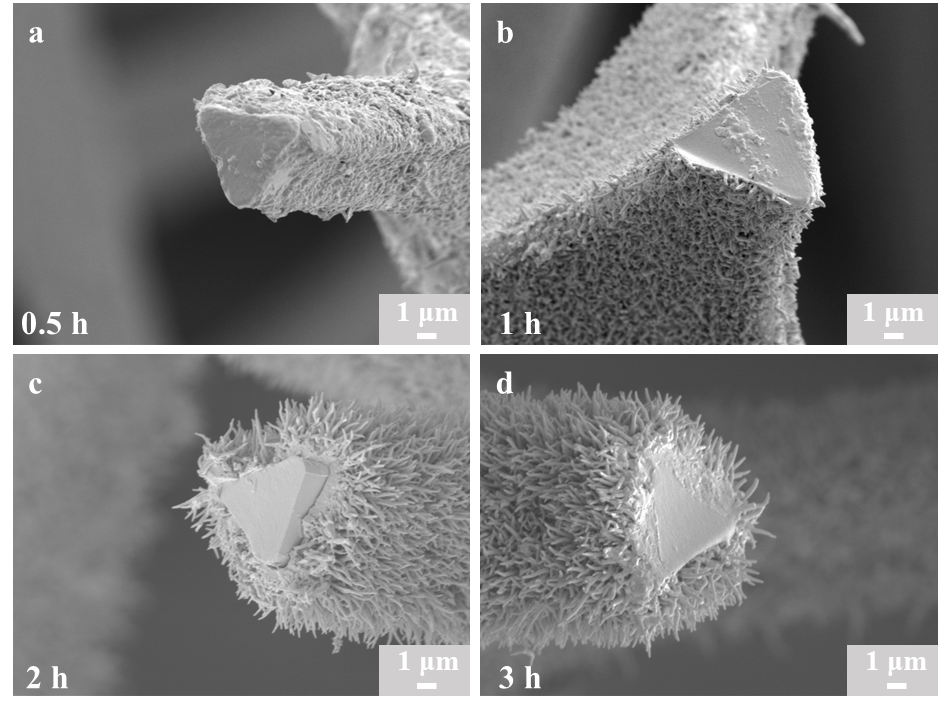


**Fig. S2** Cross-section SEM images of MF@PPy foams at different polymerization times. **a** 0.5 h, **b** 1 h, **c** 2 h, and **d** 3h


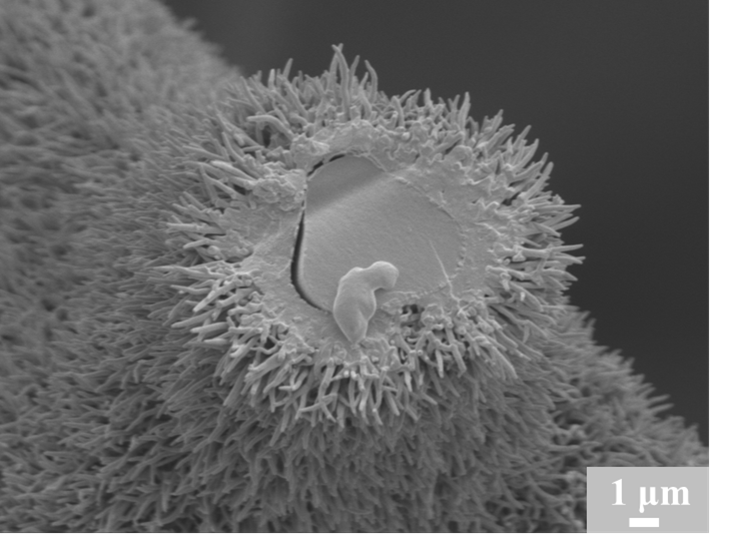


**Fig. S3** Cross-section SEM images of MF@PPy foams with PDMS

***
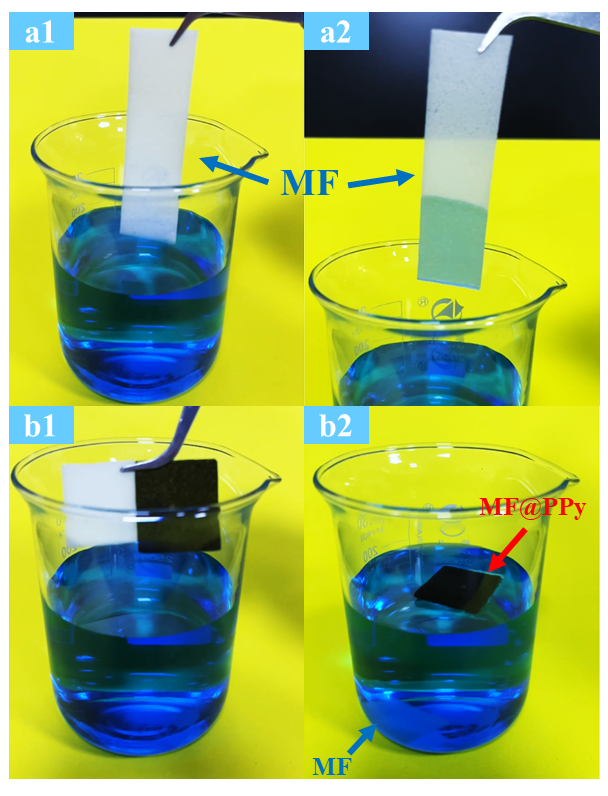
***

**Fig. S4** **a1, a2** Hydrophilicity of the pristine MF. **b1, b2** Hydrophobicity of the MF@PPy foams

**
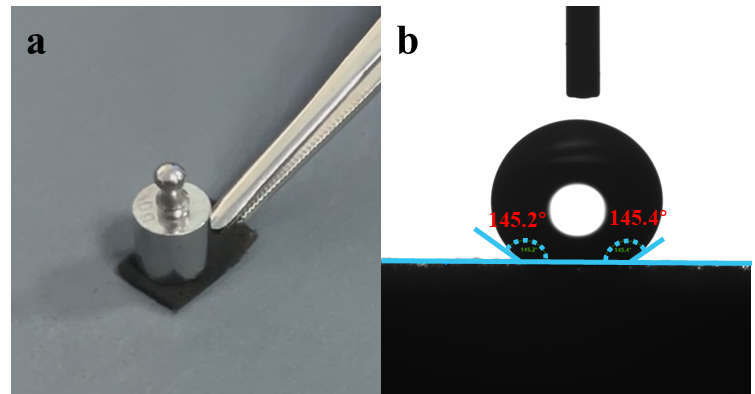
**

**Fig. S5** **a** Physical mechanical sliding of the MF@PPy foams. **b** Hydrophobic properties of the MF@PPy foams after physical mechanical sliding


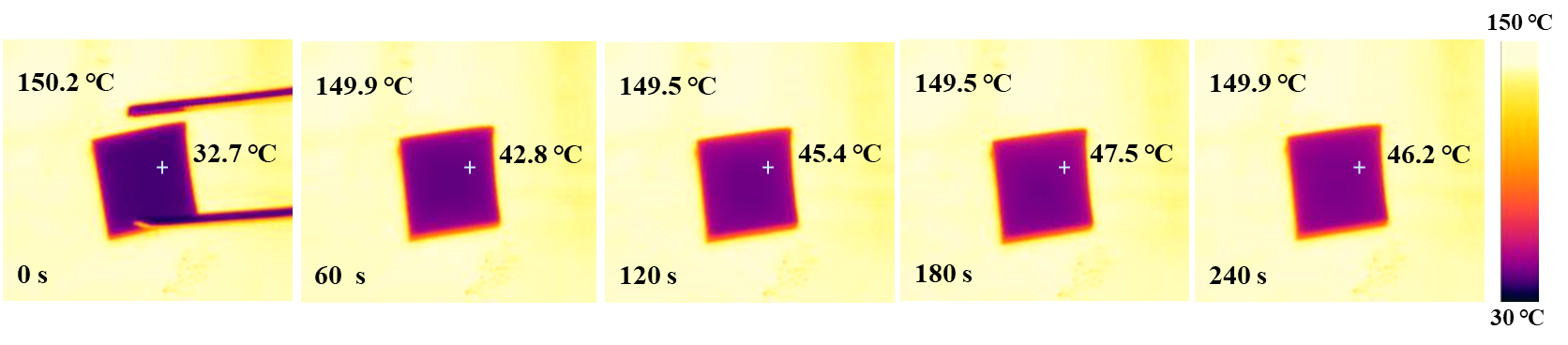


**Fig. S6** The thermal camouflage performances of MF@PPy foams at high temperatures


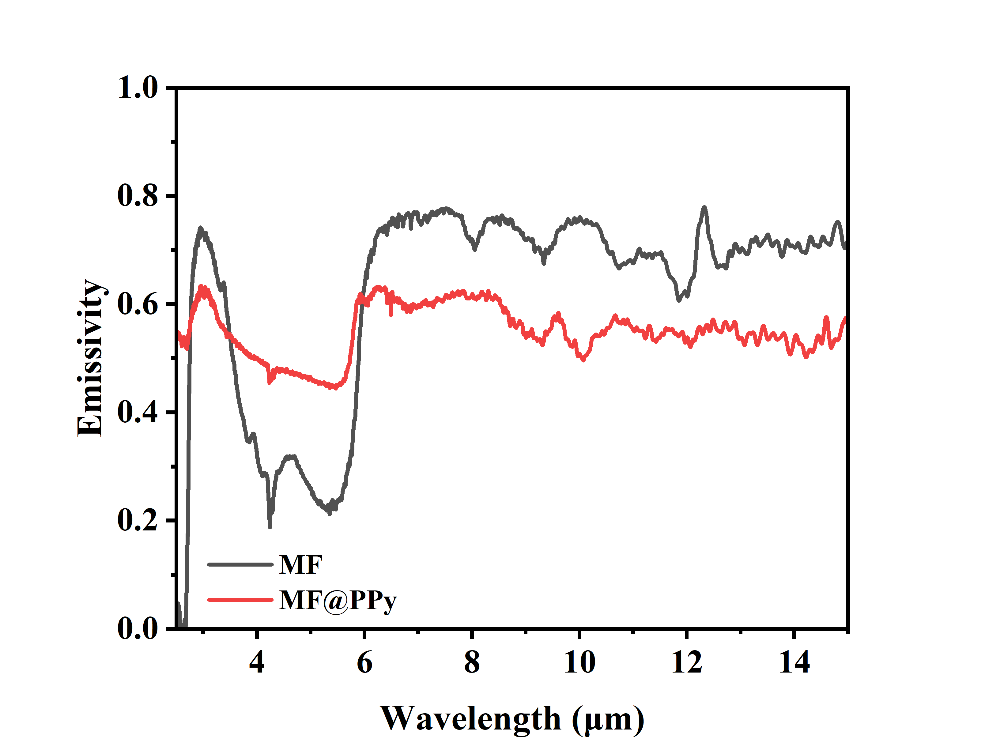


**Fig. S7** Infrared emissivity of MF and MF@PPy foams

**
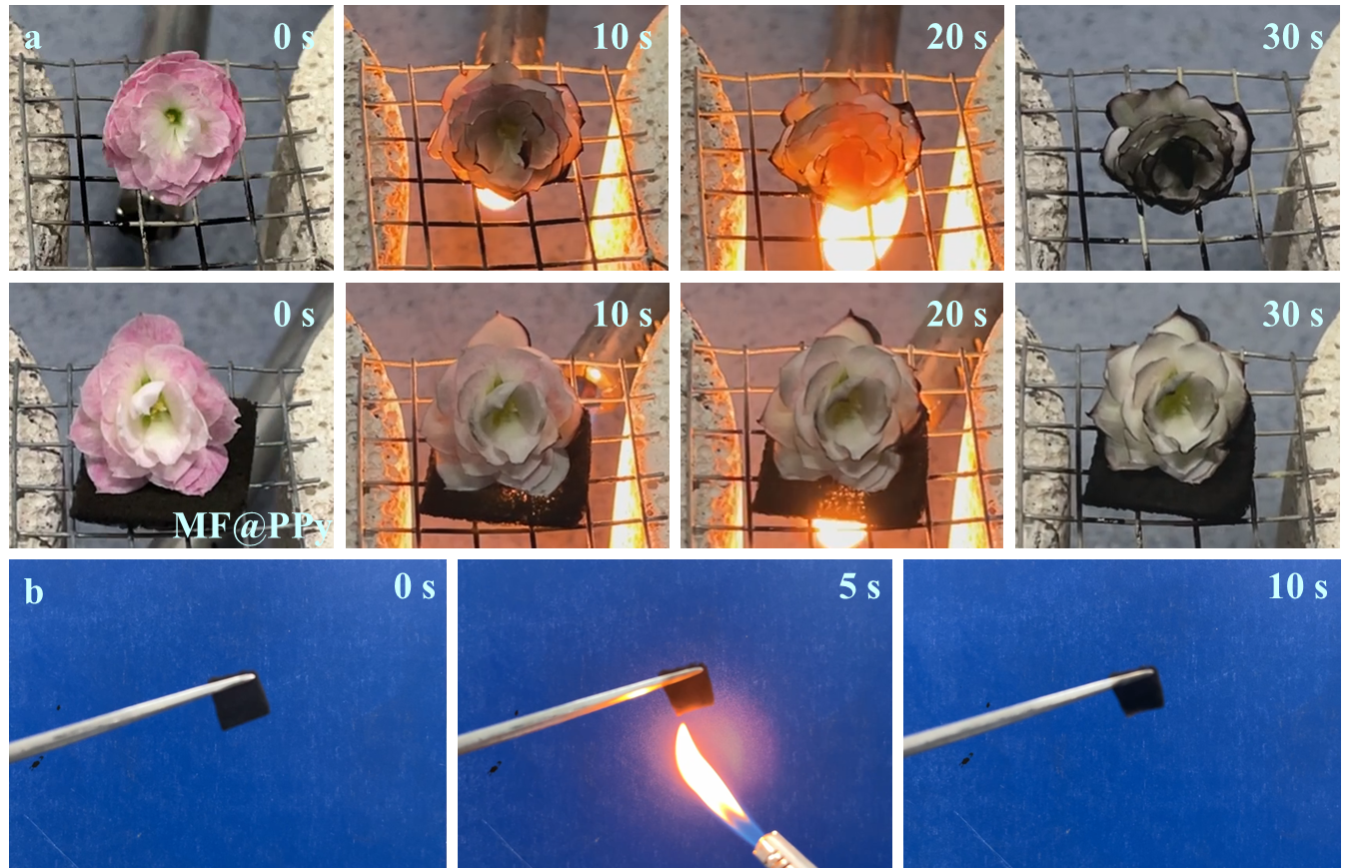
**

**Fig. S8** **a** Photographs of the flower heated by fire without or with the MF@PPy foam. **b** Flame retardant characteristics of the MF@PPy foams


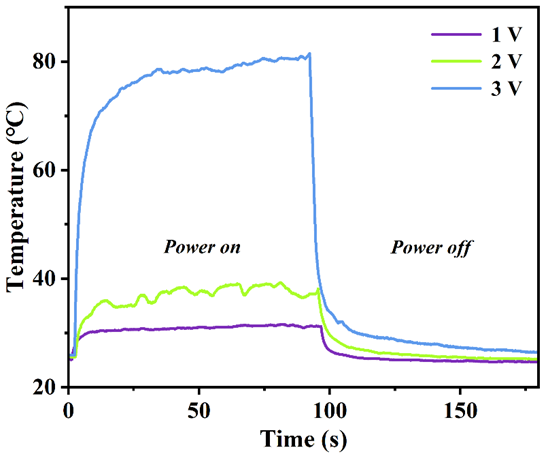


**Fig. S9** Joule heating performance of MF@PPy foams after dip-coating PDMS


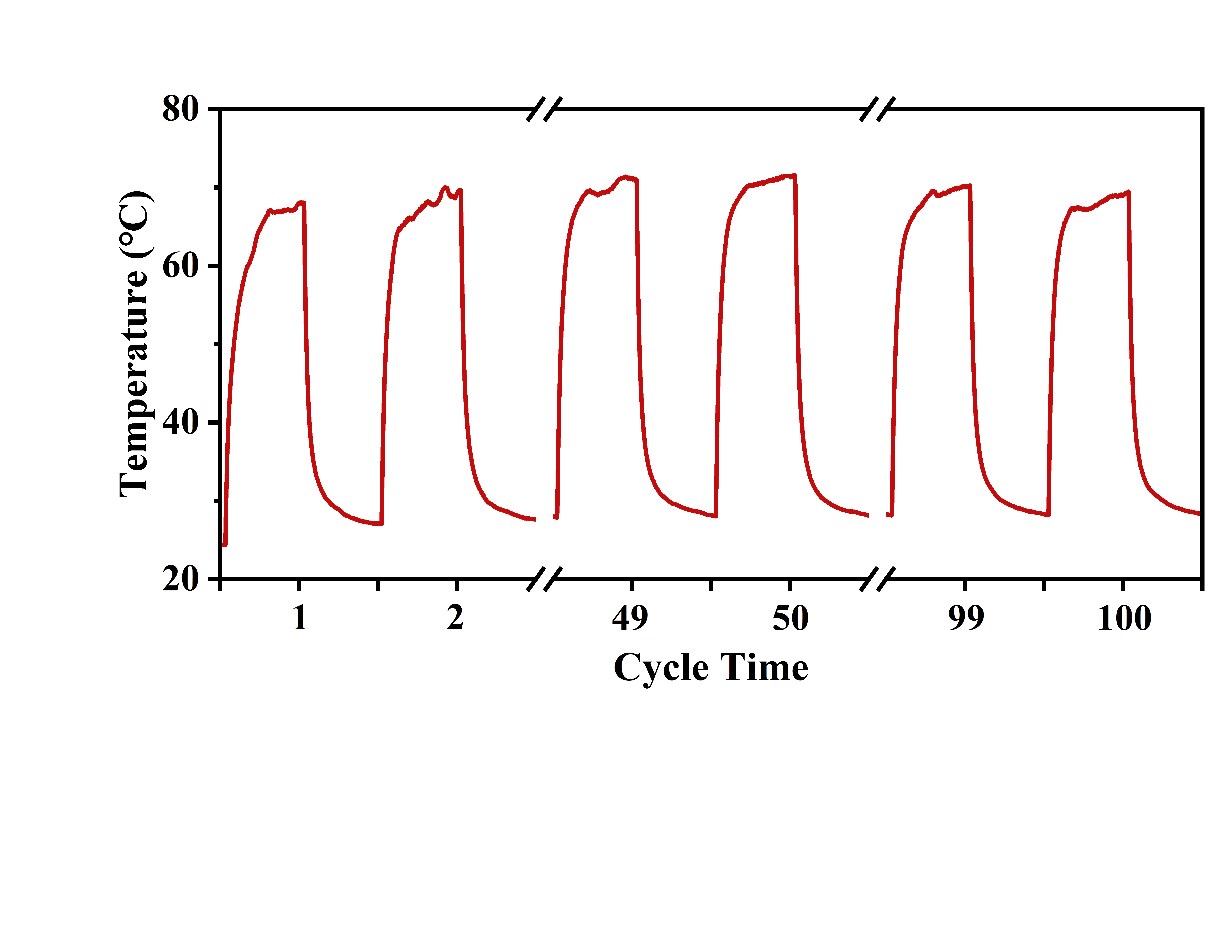


**Fig. S10** The long-term stability of Joule heating performance of MF@PPy foams


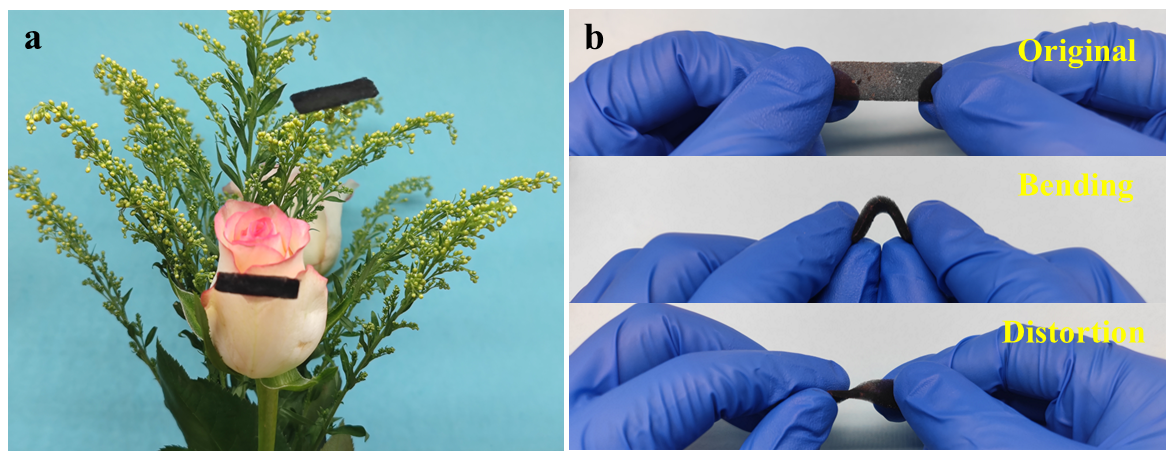


**Fig. S11** **a** Lightweight and **b** flexible properties of MF@PPy foams


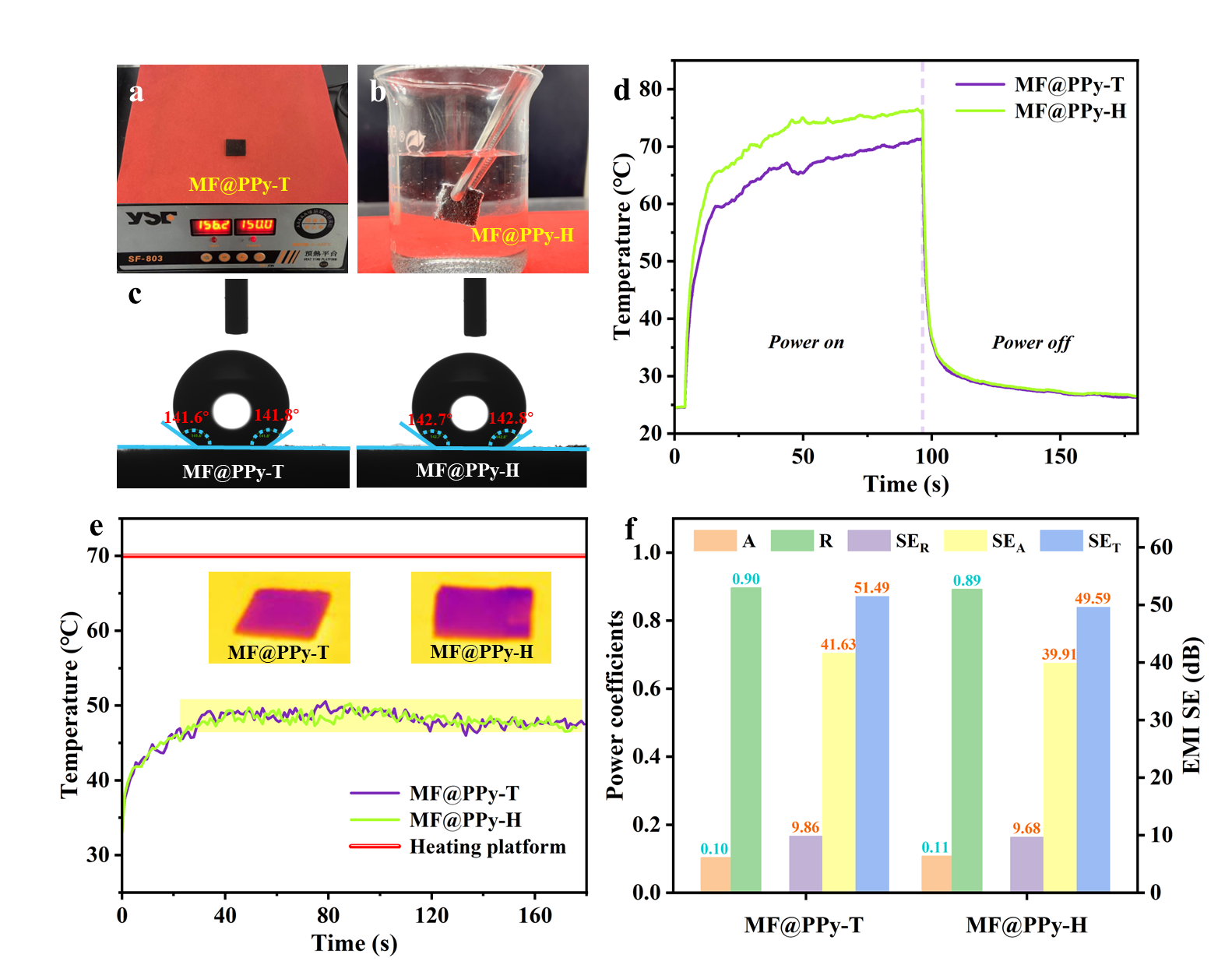


**Fig. S12** Performance of MF@PPy under **a** high temperatures and **b** high humidity: **c** hydrophobicity, **d** joule heating, **e** infrared stealth, and **f** EMI shielding
